# The relationship between gunshot-residue particle size and Boltzmann distribution

**DOI:** 10.1080/20961790.2020.1713433

**Published:** 2020-05-05

**Authors:** İlker Kara

**Affiliations:** Department of Medical Services and Technique Eldivan, Çankırı Karatekin University, Çankırı, Turkey

**Keywords:** Forensic sciences, gunshot-residue size distribution, MATLAB, SEM-EDS examination

## Abstract

Gunshot-residue (GSR) particles usually form spherical structures, have special dimensions, and a composition that consists of burned and partially unburned matter with a 0.5–50-µm diameter (sometimes larger). The GSR particle-size distributions have been argued to be caused by the effects of equilibrium-surface distributions during formation and have not yet been correlated with a theoretical analysis or probability distribution. This study proposes a model to explain the GSR particle-size distribution quantitatively. Based on the data, and, as predicted by our model, the number of GSR particles decreases proportionally to the inverse square of the GSR particle size as the particle size increases. This result occurs because of the abundance of microstructures that are encountered in the GSR particles.

## Introduction

Gunshot-residue (GSR) particles form in gas, liquid, or solid states as a result of a highly exothermic process that occurs under extreme temperature (∼2 800 °C) and high pressure (∼0.3–0.6 tons/cm^2^) [1]. Particles that form as a result of a rapid process (a few milliseconds) are scattered from the gun to the sides, forward, and backward. Particles that are scattered under an extreme temperature and a high pressure solidify by sudden cooling and reach equilibrium in various dimensional structures [2,3].

An observation of the rapid formation of GSR particles suggests that the particle-size distributions are not caused by a simple solidification. To understand this process, we wanted to know (i) whether an interaction exists between the size distributions of the formed GSR particles, and (ii) what the general behaviour of the size distribution is among different ammunition types.

To answer these questions, a detailed model for the GSR particle sizes needs to be prepared by a quantitative analysis. Here, a quantitative analysis may explain the particle-size distribution and dimensional characteristics [4–7]. We are developing an approach that shows the general behaviour of the size distribution of the GSR particles. The proposed approach is based on the solidification assumption, which states that GSR particles are divided into smaller pieces to become stable (including fragmentation that is caused by the mechanical system). As a result, the dimensional structures of the GSR particles consist of a balanced, stable, and sustainable system that is created by particle division or integration [8].

**Figure 1.**
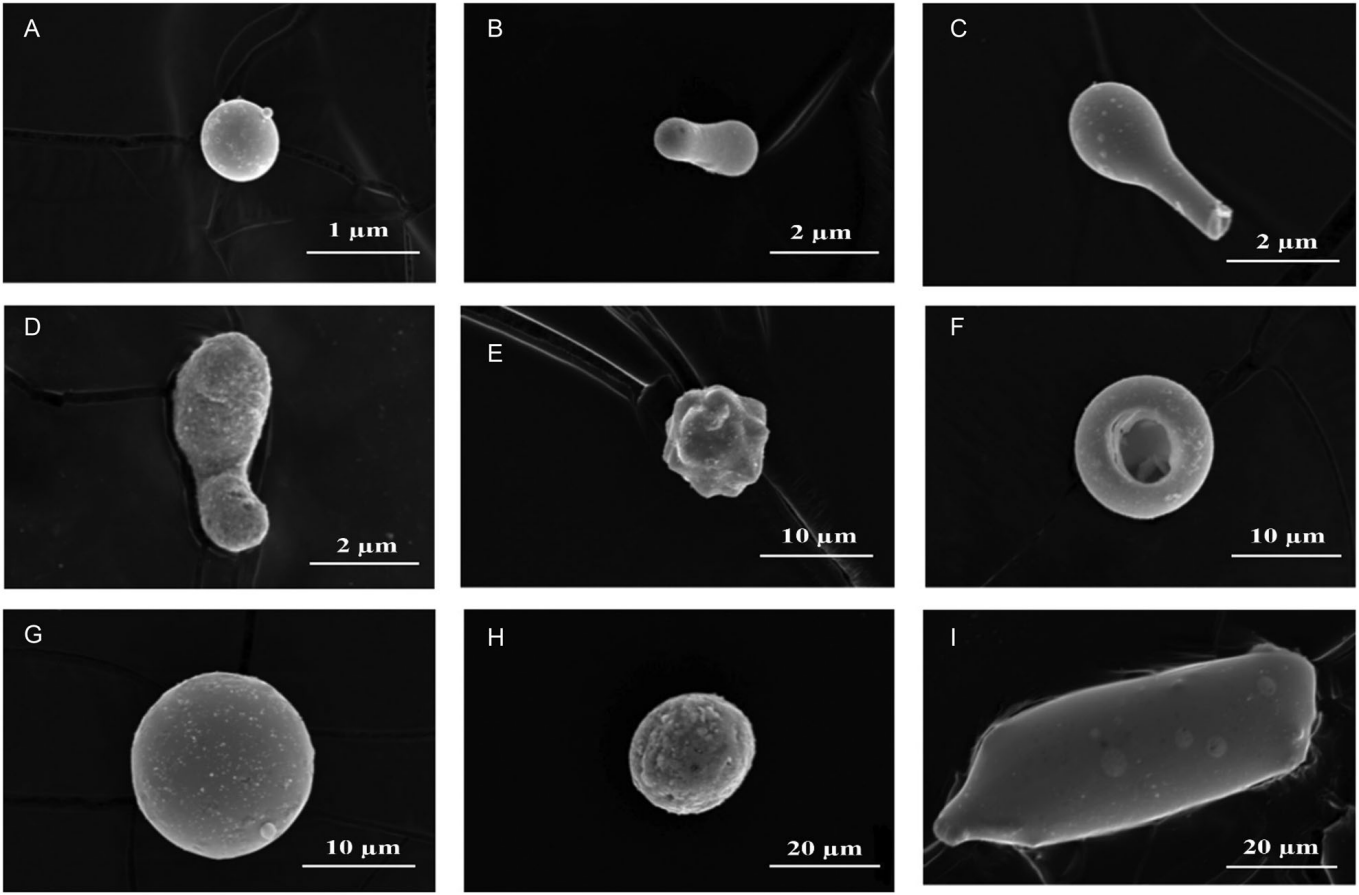
Gunshot-residue (GSR) particles of different sizes formed by fragmentation–splitting and external factors. (A, G, H) spherical structure; (B, D) structure formed by merging; (C, F) structure formed by fragmentation; (E, I) irregular structure.

Because of the extreme temperature and high pressure, the GSR particles exist in liquid state initially. Excess energy and unbalanced forces affect the GSR particle droplets. The GSR particles in the liquid state solidify as spherical droplets with the smallest surface area in the same volume to reach a minimum energy level. The best example of this case is the perfect sphere (Figure 1A).

If the adhesion forces between the surfaces of the liquid GSR particles are sufficiently strong, these forces bind and hold the particles together, which results in macrostructures (Figure 1I). The GSR particles in the liquid state solidify in a sustainable equilibrium through splitting and merging. The melting temperature of the constituent elements is one of the most critical factors in the formation of a dimensional structure of the GSR particles that are formed in this observed equilibrium [9–11].

Literature studies [12–17] confirm that the dimensional structure of the GSR particles consists of spherical particles as a result of a random distribution. Vermeij et al. [10] showed that differences may exist in the dimensional structures of the GSR particles outside of the spherical structures. Brozek–Mucha [18] classified GSR particles according to their dimensional structures for the first time. In later studies, Brozek–Mucha [19] examined the distribution of the dimensional structures of GSR particles according to their chemical content.

**Figure 2.**
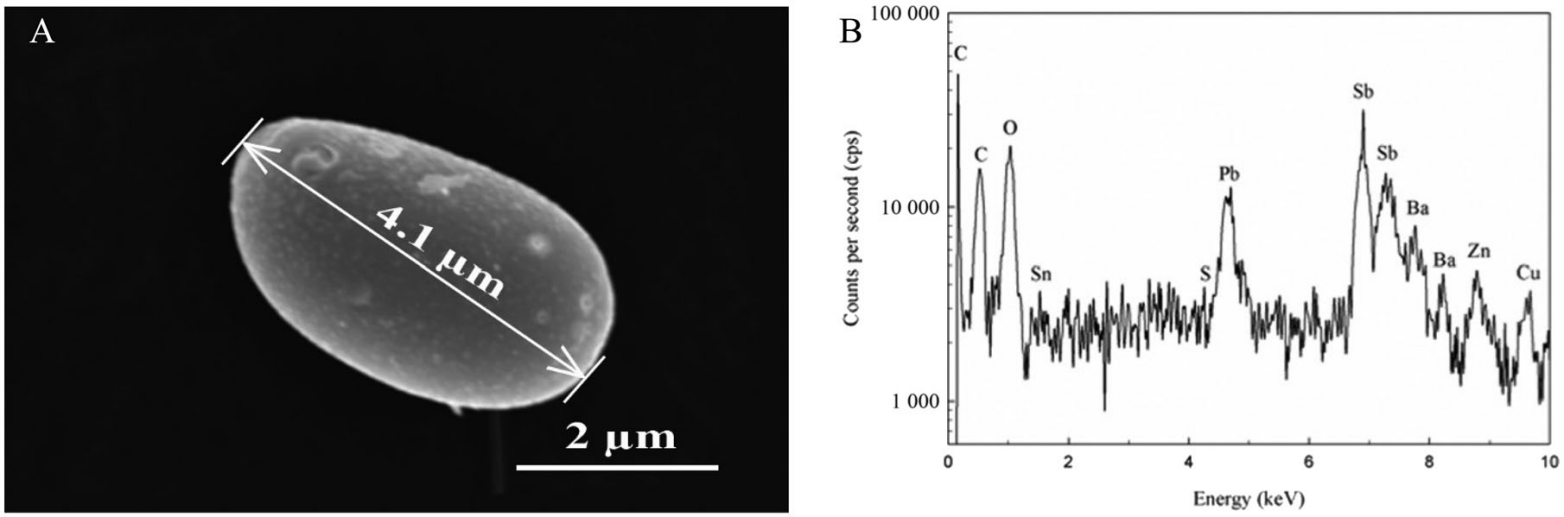
Gunshot-residue (GSR) particle-size measurement (A) and energy dispersive spectroscopy (EDS) spectrum (B).

In GSR analysis, forensic experts make decisions by looking at the chemical and structural characteristics of suspicious samples (Figure 2) [14,15]. The GSR structure tends to be spherical and analyses focus on the existence of such structures in suspicious samples [13–15]. Therefore, a determination of the structural distributions of the GSRs will help forensic experts in their GSR analysis. This study focuses on the argument that the dimensional size distribution of GSR particles can be explained by a specific distribution function. For this purpose, GSR particles that formed according to structure sizes and numbers were classified and analysed according to distribution functions. The Boltzmann distribution is the most basic distribution principle for a system and refers to a given distribution function or probability measurement [20,21]. The dimensional size distribution of the GSR particles was analysed according to the Boltzmann distribution function. The study showed that the dimensional distribution of the GSR particles is not a random distribution, but was found to follow the Boltzmann distribution function.

## Materials and methods

All tests were carried out at the Criminal Police Laboratory (KPL in Turkish) shooting room. In the test shots, a 9-mm Sarsilmaz Kilinc 2000 mega brand semiautomatic pistol was used with full metal jacket cartridges that were produced by a 9 mm × 19 mm Parabellum-type MKE, Geco, S&B, WIN, and LIBRA. The sample were collected on double-sided adhesive tape glued to aluminium stubs from shooter's right hand for analysis by scanning electron microscopy (SEM). Three shots were fired with each ammunition type. GSR was collected by pressing the stub 30 times to skin on the hand, including the thumb and index finger of the right hand of the shooter. The residues were collected from the shooter’s right hand and analysed with the same strict conditions. This experimental study was conducted in the department of KPL. In the tests, the weapon barrel was cleaned before each shot. The cleaning process started with mechanical cleaning. Then, the barrel was washed in an ultrasonic bath of ethanol and deionized water before being dried with dry nitrogen gas. The collected GSRs were examined using a JEOL/JSM-6400 SEM (JEOL Ltd., Akishima, Japan). The acceleration voltage was 20 kV, the tilt of the sample was 0°, and the working distance was 39 mm. Images were secondary electron images coupled with an INCA energy X-ray spectrometer (Oxford Instruments Ltd., Houston, TX, USA).

### Significance

Although the GSR particle-size distribution is assumed to be random, it remains uncertain whether this observed distribution is unique and universal [22]. Because the dimensional structure formation and GSR particle-size distributions are not known in detail, the results of this study may be an outline for all GSR particles [23]. The dimensional size distribution of the GSR particles decreases proportionally to the inverse square of the number of GSR particles that formed as the particle size increased. For this reason, the same size distribution is expected compared with the different types of ammunition analysed. This expectation may result from similarities in the formation mechanisms of the GSR particles (including the particle size: fragmentation–merging and external factors, air resistance and fragmentation caused by the mechanical system).

## Results and discussion

### Dimensional analysis

To understand the qualitative behaviour, it is instructive to start with the simplest model that allows for an analytical treatment [24].

The dimensional classes can be obtained using the sizes (r_1_) of the primary particles of the GSR and the total number of particles (m_1_). The density of the primary class r_*k*_ (*k* = 1, 2, 3 …) can be determined from r_*k*_ = r_1_*k*^1/2^. The classification distribution that was obtained for all particles can be described by the Enskog–Boltzmann theory. In this case, the dimensional distribution ratio of the GSR particles depends on splitting–merging and external factors. In fragmentation that is caused by exposure to air resistance and a mechanical system under an extreme temperature and high pressure, the external factors may cause a deviation in the dimensional distribution. Collisions among GSR particles and with the mechanical system can cause fractures and fragmentation in the dimensional structure (Figure 1).

Table 1 shows the statistical distributions of GSR particles of five different types of ammunition according to their dimensional characteristics. They were analysed in nine groups according to their dimensional characteristics. Of the five types of ammunition, small structures (0–1 µm) were the most frequently seen dimensional group. Although the number of macrostructures that was formed by a combination of small structures was smallest, the small structure was the most observed dimensional group in the S&B brand ammunition. Figure 1 shows samples of dimensional forms of GSR particles of five different ammunition types, detailed in Table 1. Figure 1(A, G) MKE brand; (B, C) Geco brand; (H, D) WIN brand; (E, F) LIBRA brand; (I) S&B brand ammunition selected randomly.

**Table 1.** Statistical values for investigated particles with different dimensional properties from five types of ammunition.

	Amount of particles														
	Ammunition type (9 mm); mean value $\bar{X}$					Standard deviation					Confidence range				
Size of particles (μm)	MKE	Geco	S&B	WIN	LIBRA	MKE	Geco	S&B	WIN	LIBRA	MKE	Geco	S&B	WIN	LIBRA
0–1	131	149	105	93	85	35.7	29.2	17.5	13.3	11.8	23–284	37–279	40–190	28–113	34–136
1–2	54	46	38	33	29	12.0	7.5	5.7	4.8	3.6	2–102	1–65	2–56	12–45	13–44
2–3	30	25	24	20	19	3.1	5.1	4.4	3.2	2.9	16–46	3–56	5–43	5–32	7–31
3–5	19	22	15	12	10	2.8	2.8	2.2	1.8	1.4	7–26	10–34	6–24	3–17	4–14
5–10	15	18	11	9	7	2.2	1.9	1.2	0.9	0.7	5–20	10–26	6–16	4–10	4–10
10–15	8	9	7	5	4	1.7	0.9	0.6	1.7	0.5	1–14	5–14	4–10	1–15	2–6
15–20	6	7	5	4	5	0.9	1.1	0.3	0.8	0.9	2–8	2–12	4–6	3–9	1–8
25–35	4	3	7	4	3	0.4	0.3	0.5	0.5	0.7	2–6	2–4	5–9	2–6	1–5
>35	2	2	6	3	4	0.2	0.3	0.5	0.3	0.5	1–3	1–3	4–8	1–3	2–6

MKE: Turkey production; Geco: German production; S&B: Czech Republic production; WIN: US production; LIBRA: Czech Republic production.

In the proposed model, although the GSR particle sizes and amounts have different values, the size distribution of the dimensional classes agreed with the inverse square method (Boltzmann distribution).

These results are based mainly on three underlying assumptions:

- a merging with the effect of adhesion forces,
- a split by excess surface energy and the effect of unbalanced forces,
- fragmentation because of external factors (such as air resistance and a mechanical system under extreme temperature and high pressure).

The particle sizes that are formed are determined by these effects.

### Size distribution of GSR particles

#### Boltzmann distribution

The basic equation for the number of molecules with variable velocities and energies is found from the Boltzmann distribution [21, 25]. According to the model based on kinetic molecular theory, gas molecules move at various velocities and in various directions. The distance of each molecule to its starting point is proportional to the magnitude of the molecular velocity, and the molecule behaves isotropically because the distribution of molecular velocities in all directions is the same. The velocity distribution in a direction, for example in the *x*-direction, can be calculated. For molecules in the velocity range of *u_x_* and *u_x_* + *du_x_*, the $\frac{dN}{N}$ fraction needs to be found.

For the fraction of molecules in this velocity range, the following is used:

$$\frac{dN}{N}=Ae^{-\frac{1}{2}mu_{x}^{2}/kT}{\mathrm{du}}_{x}$$

where *A* is the proportion constant. The value of this constant can be calculated from the integral of the right side of the equation. All possibilities for *u_x_*, which is the velocity in both directions (positive *x*-direction and negative *x*-direction), will be in the range *u_x_* = ±∞. For this reason, $\frac{dN}{N}$ = 1, exists in this velocity range.

$$\int_{-\infty }^{+\infty }Ae^{-\frac{1}{2}mu_{x}^{2}/kT}{\mathrm{du}}_{x}=1,\mathrm{hence}$$

$A=\frac{1}{\int_{-\infty }^{+\infty }Ae^{-\frac{1}{2}mu_{x}^{2}/kT}{\mathrm{du}}_{x}}$ can be used for the A constant. The value of A in this equation can be calculated as $A=\sqrt{\frac{m}{2\pi kT}}$. As a result, $\frac{dN}{N}=\sqrt{\frac{m}{2\pi kT}e^{-\frac{1}{2}mu_{x}^{2}/kT}}$ can be used for the velocity distribution of N molecules in the *x*-direction.

In summary, in the Boltzmann distribution, velocities of various molecules at the same temperature vary inversely proportionally to the square root of the molecular masses. As the molecules become smaller, their velocities increase. This result conforms to the Graham law, which is found experimentally. In this study, experimental data were analysed in a simplified system, and then divided into groups. The distribution of GSR particle sizes tends to be found to be consistent with the distribution that was obtained by the Boltzmann kinetic equation [26,27]. This result may occur because of the solidification of the GSR particles that reached equilibrium in structures that were smaller than a certain size (average 0–1 µm).

**Figure 3.**
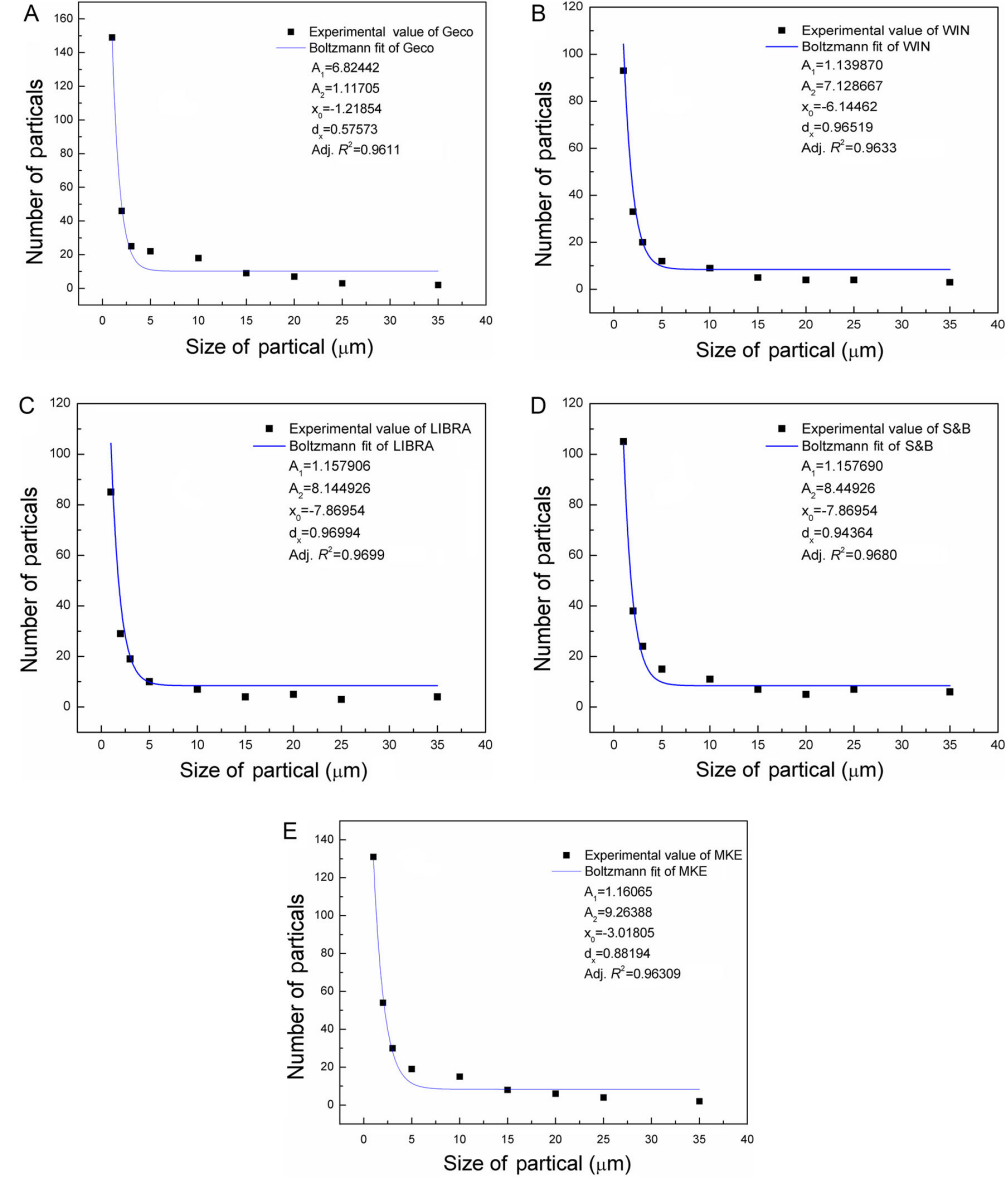
Fit curve of variation of Gunshot-residue (GSR) particle size–particle number values obtained from Geco (A), WIN (B), LIBRA (C), S&B (D), and MKE (E) brand ammunition according to the Boltzmann distribution.

The size distribution of the GSR particles is a mathematical result that is obtained by the Boltzman distribution in accordance with the above assumptions. An analysis of the data was performed by using the MATLAB computer programme (The MathWorks, Inc., Natick, MA, US). Plots that were obtained using the MATLAB programme are shown in Figure 3. Using the MATLAB programme, the model curve that fits the experimentally obtained data can be identified. The data in the intervals that are classified as the result of the analysis showed a behaviour that conformed to the inverse square method (Boltzmann distribution).

## Conclusion

In this study, a model was developed for the GSR particle-size distribution, which occurs in a dynamic equilibrium between the splitting–merging mechanisms and external factors. The proposed model was analysed using the MATLAB programme and the results obtained were presented in detail. The model explains the particle-size distribution properties of GSR by quantitative analysis. The results of this study may provide an outline for all GSR particles. It is necessary to improve the studies further to enable a comparison of different brands and scale of ammunition.

In experimentally observed physical formations of GSR particles, it is believed that the mechanical system and the air resistance that was exposed during the scatter were effective, as were the formations because of splitting–merging. When the GSR particle-size distributions are examined, the dominance of the microstructures (particles from 0 to 1 µm) suggests that split-fragmentation mechanisms are dominant. Although the number of macrostructures that was formed by the merging mechanism was small, structures existed with dimensions that exceeded 50 µm. GSR particles that transform into liquid form under high pressure and extreme temperature are under the influence of unbalanced forces because of the excess surface energy. To reduce this effect and achieve stabilization, GSR particles form as small surfaces as possible and solidify with sudden cooling, which results in microstructures. If the adhesion forces between the surfaces of the GSR particles, before they solidify with sudden cooling, are sufficiently large, the particles solidify into macrostructures. These results show that the number of macrostructures is low compared with the microstructures.
